# Supplementary material for: Evaluation of MoveSmart MS - an online structured exercise, Social Cognitive Theory-based behavioural coaching and peer support programme - on anxiety in multiple sclerosis
Source: PLoS One. 2025 Nov 10;20(11):e0336493. doi: 10.1371/journal.pone.0336493 (PMC12599958; doi:10.1371/journal.pone.0336493)
Supplement: S2 File — (PDF) [file pone.0336493.s002.pdf]

S2: CHAIR'S DECISION RESEARCH ETHICS APPLICATION FORM

UNIVERSITY OF LIMERICK
OLLSCOIL LUIMNIGH
Education and Health Sciences

**Title of Project**	**Move Smart MS**
**Related Approval Code**	**2020-12-19**
**Period for which Approval is Sought**	**To December 2024**
**Principal Investigator (and qualifications)**	**Name:** ***Please change to Prof Amanda Clifford as Dr Toomey has left UL***<br>**Department: SAH**
**Other Researchers (and qualifications)**	**Name/s:** Susan Coote PHD, Laoise O'Driscoll and Olivia Carrington, MSc Physio Prof Qual students, Dr Nicola O'Malley, and Tina O'Connor, Senior Physiotherapist MS Ireland<br><br>***Please add Hugh O'Loughlin Senior Physiotherapist MS Ireland***<br><br>***Please add Dr Rebecca Maguire and Austin Fahy, Maynooth University.***

Please provide a description of the proposed changes to the previously approved research under the following headings.

1. **Research Question/Hypothesis**

We propose asking new questions of the qualitative and quantitative data:

- Did anxiety symptoms change following participation in the Move Smart Programme?
- To what extent can (1) sociodemographic factors, (2) health status and experience of MS symptoms, and (3) psychological and lifestyle factors predict anxiety levels at baseline?
- To what extent can the above factors predict changes in anxiety after participation in the Move Smart programmes?
- What is the experience of peer support in the context of the reduction of anxiety as a result of this programme?

2. Research Design

Analysis of qual data using Reflexive Thematic Analysis. Analysis of RCT data using moderator and mediator analysis.

3. Data Collection Methods

No change

4. Subject Number & Selection Procedures

No change

<table>
<tr><td colspan="3">5. Changes to Supporting Documentation

No change</td></tr>
<tr><td colspan="3">6. Other Changes</td></tr>
<tr><td>Signature of Principal Investigator:</td><td></td><td>Date:</td></tr>
<tr><td>Signature of Head of Department</td><td></td><td>Date:</td></tr>
</table>

MS Ireland
THE MULTIPLE SCLEROSIS SOCIETY OF IRELAND

UNIVERSITY OF LIMERICK
OLLSCOIL LUIMNIGH
Education and Health Sciences

MOVESMART MS
SPECIALIST ONLINE EXERCISE PROGRAMMES

VOLUNTEER INFORMATION SHEET

Move Smart MS, Interview Study

Dear participant,

You have recently taken part in an online physiotherapy programme which is part of the Move Smart MS project. We want to learn more about your experience by interviewing you with participants from other programmes.

What is the study about?
The study aims to find out what you thought about the Move Smart MS programme you took part in.

What will I have to do?
You will be invited to take part a Zoom meeting at a time that suits you along with 5-6 participants from other Move Smart physio groups. The focus groups will involve all of you logging on to a Zoom meeting in a similar way that you did for your class, and being asked questions about the programme, how you found it, what difference it made, whether you are continuing with the exercises and suggestions for improvements. The Zoom meeting will be video recorded.

What are the benefits?
The findings of the study might help MS Ireland deliver more effective programmes, and it might help us lobby for future funding for programmes. You may learn more about other peoples experiences and learning from their programmes.

What are the risks?
You might decide that you don't want to answer a question. If this happens, you do not have to answer any question you do not wish to.

What if I do not want to take part?
Participation in this study is voluntary and you can choose not to take part or to stop your involvement in this study at any time.

What happens to the information?
The information that is collected will be kept private and stored securely and safely on the researchers' computer. The computers are protected with a password and encrypted. Your

name will not appear on any information. You will be assigned a fictitious name when the information is being written in a report by the researcher. The information that is gathered in the study will be kept for seven years. After this time, it will be destroyed. The videos will be deleted once we have transcribed what has been said in the focus groups.

Who else is taking part?
Other people with MS who took part in Move Smart MS physio programmes. There may be some people from your own group but more than likely there will be people you don't know who have taken part in other programmes.

What if something goes wrong?
In the unlikely event that something goes wrong during the focus group session, the interview session will immediately stop until the researcher and student(s) are ready to restart the session or the session would be stopped completely.

What happens at the end of the study?
At the end of the study the information will be used to present results both for research and service planning purposes. The information will be completely anonymous. Your name will not appear in any of the results. All data gathered from the research will be stored securely and safely by Susan Coote in MS Ireland or in UL on a computer that is password-protected for 7 years.

What if I have more questions or do not understand something?
If you have any questions about the study you may contact either of the researchers. It is important that you feel that all your questions have been answered.

What happens if I change my mind during the study?
At any stage should you feel that you want to stop taking part in the study, you are free to stop and take no further part. There are no consequences for changing your mind about being in the study.

<u>Contact name and number of Project Investigators.</u>

Principal Investigator
Dr Elaine Toomey, School of Allied Health, University of Limerick
Email: Elaine.C.Toomey@ul.ie

Other investigator
Dr Susan Coote
MS Ireland
Tara House,
Limerick
Email; SusanC@ms-society.ie

This research study has received Ethics approval from the Education and Health Sciences Research Ethics Committee (2020-12-09)
If you have any concerns about this study and wish to contact someone independent you may contact:
Chair Education and Health Sciences Research Ethics Committee
EHS Faculty Office
University of Limerick
Tel (061) 234101

FACULTY OF EDUCATION AND HEALTH SCIENCES

RESEARCH ETHICS COMMITTEE (EHSREC)

Ethical Consent Form

I, the undersigned, declare that I am willing to take part in research for the project entitled

"*Move Smart MS – qualitative study*".

- I declare that I have been fully briefed on the nature of this study and my role in it and have been given the opportunity to ask questions before agreeing to participate.
- The nature of my participation has been explained to me, and I have full knowledge of how the information collected will be used.
- I am aware that my participation in this study will be video recorded and I agree to this. However, should I feel uncomfortable at any time, I can request that the recording software be switched off.
- I am aware that such information may also be used in future academic presentations and publications about this study.
- I fully understand that there is no obligation on me to participate in this study.
- I fully understand that I am free to withdraw my participation without having to explain or give a reason, up to a period of two weeks after the data collection is completed.
- I know that I have been asked not to discuss the content of the focus group discussion, or the identity of its participants with anyone.
- I acknowledge that while the researcher has asked all focus groups participants to maintain confidentiality in the above manner, the researcher cannot guarantee that individual participants will adhere to this request.
- I acknowledge that the researcher does guarantee that they will not use my name or any other information, that would identify me in any outputs of the research.
- I declare that I have read and fully understand the contents of the Research Privacy Notice.

____________________________________ _________________________

Signature of Participant Date

____________________________________ _________________________

Signature of Investigator Date

I explicitly consent to the University contacting me as part of current or similar future research and holding my contact details on its database for the purpose of contacting me.

Yes ❐ No ❐
